# Case of Perforation of the Stomach

**Published:** 1829-03

**Authors:** James Leonard

**Affiliations:** Member of the Royal College of Surgeons, London.


### PERFORATION OF THE STOMACH.

*Case of Perforation of the Stomach.* By James Leonard, Esq. Member of the Royal College of Surgeons, London.

Wm. Shepherd, aged twenty-six, by trade a stonemason, was leaving the quarry at six p.m. when he suddenly laid his hand upon his left side, uttered a groan, and fell. I found him with a face indicating extreme anxiety, livid lips, his knees drawn up towards his belly, which, owing to the contraction of the muscles, was as hard as a board; his breathing short; his nostrils dilated; his pulse 130, small and thready; urgent thirst; cold extremities, and violent pain diffused over the whole abdomen, but most acute in the left hypochondrium.

On examination, I found no hernia, but was informed that his bowels were constipated, the last alvine evacuation having taken place three days before.

Being of opinion that it was a case of peritonitis, I opened a vein on the spot, but got only twelve ounces of blood.

Upon his removal to his house, however, I took thirty ounces more, and his pulse became fuller. A warm bath was prepared, and after he had been in it ten minutes the pain abated. As soon as he was taken out of the bath, a large vesicator was applied to the abdomen, and an ounce of Oleum Ricini was given to him, which he had scarcely swallowed when he retched violently, but rejected nothing. The pain in the abdomen rapidly increased, with excessive thirst, and dreadful restlessness. About midnight I took twenty ounces more of blood. Enemata of various composition, chiefly emollient, were administered during the night, without effect. His belly was excessively tense, and he had drunk much, but vomited nothing, although the retching was severe.

At four A.M. his pulse began to intermit, and at six was imperceptible; and, while grasping the vessel to take another draught, he fell back and expired.

Twenty-four hours after death the body was inspected, and exhibited the following appearances. On opening the cavity of the peritoneum, the first thing that presented itself was the Oleum Ricini, floating on the surface of a large quantity of turbid fluid, amounting to not less than three or four quarts. The peritoneum was highly vascular, and large patches of a gangrenous appearance were visible on several parts of it. From the intestinal portion of that membrane much coagulable lymph had been thrown out, so as to fill up the spaces between the convolutions, and produce the appearance of a continuous surface, which was easily broken down. The omentum was equally vascular. On raising the left lobe of the liver, a small orifice was visible in the anterior surface of the stomach, about two inches from the pylorus. At the point of perforation the stomach was an inch thick. The diameter of the tumor was about an inch and a quarter. The external orifice was perfectly round and well defined, as if stamped out, and would have admitted a pea. The internal opening was ragged, and surrounded with a yellowish pus. The stomach was quite empty, and had no adhesion to the liver at the place of perforation. With the exception of great vascularity, produced by the irritating substances admitted into the cavity of the peritoneum, the other viscera were healthy.

The deceased had, from the age of sixteen to that of twenty-two, been much addicted to the use of undiluted ardent spirits; but, for four years previous to his death, having been troubled with symptoms of dyspepsia, he had been advised to live more temperately. With this advice he had complied and was much better; but occasionally there was a return of the acute pain under the ensiform cartilage, accompanied with vomiting, which seldom lasted above four or five hours, and always yielded to an anodyne draught, followed up by some mild laxative medicine.

Having read Dr. Ebermayer's cases, recorded in Rust's Magazine,* I find that this case coincides, in most of its features, with his: viz. that it was of a chronic nature; that there were scarcely any previous symptoms indicating extreme danger; that the powers of digestion were but very slightly affected by the disease; that there was no emaciation, although his countenance had not the ruddy appearance of health which would have been expected from the muscularity of his body.

In this case, however, the remote cause would appear to have been *long-continued irritation* from the stimulus of ardent spirits. Perhaps the stomach, having been so long accustomed to the stimulus, might have suffered a diminution in the secretion of gastric fluid when it was left off; but the disease existed before that. And his opinion that in no case had any traces of inflammation or ulceration been observed, is not in accordance with what this case demonstrates; for it was evidently the result of a long-continued process of chronic inflammatory action and ulceration, yellow pus being found in the wound. In other respects the cases are similar. Wherever the disease occurs, it must prove fatal, and no peculiar symptoms mark the case till too late for human aid to be of any avail.

6, *St. Martin's street; Feb. 7th,* 1829.

---

### ANIMAL MAGNETISM.

*On Mesmerism, improperly denominated Animal Magnetism.* By Richard Chenevix, Esq. F.R. and E.S. M.R.I.A. &c.†

Animal magnetism is true. In the whole domain of human acquirements, no art or science rests upon experiments more numerous, more positive, or more easily ascertained. As this assertion is in direct contradiction to the

* We gave a translation of these cases in our Number for October, 1828.—Editors.

